# Secondary Metabolism Gene Diversity and Cocultivation toward Isolation and Identification of Potent Bioactive Compounds Producing Bacterial Strains from Thailand's Natural Resources

**DOI:** 10.1155/2022/2827831

**Published:** 2022-05-29

**Authors:** Suranat Phonghanpot, Faongchat Jarintanan

**Affiliations:** ^1^Biochemistry Unit, Department of Biomedical Science, Faculty of Sciences, Rangsit University, 52/347 Muang Ake, Phaholyothin Road, Lak Hok, Muang, Pathum Thani 12000, Thailand; ^2^Faculty of Medical Technology, Rangsit University, 52/347 Muang Ake, Phaholyothin Road, Lak Hok, Muang, Pathum Thani 12000, Thailand

## Abstract

Thailand was proposed to be rich unexplored source of microorganisms, especially bacterial strains. There should be bacteria with high secondary metabolite production potential in the natural resources that are still unidentified. Moreover, they might not produce secondary metabolites in standard laboratory culture condition after isolation, in which coculture condition would help us pursuing the bacteria to produce bioactive metabolites. Here, we aimed to identify new bacterial strains with high secondary metabolite production potential from Thailand's natural resources. To achieve the goal, we performed bacteria isolation, phylogenetic analysis, degenerate PCR of secondary metabolism genes, cocultivation, antibacterial analysis, and HPLC chemical profiling. We isolated distinct 40 bacterial strains, which have over 98% 16S rRNA sequence similarity with known species. There were 22, 31, and 29 strains giving positive PCR amplification of NRPS, PKS, and TPS genes, respectively. Among them, *Bacillus licheniformis* RSUCC0101 had the highest number of PCR products, 26. In standard single culture condition, crude extracts prepared from *Bacillus safensis* RSUCC0021 and *Bacillus amyloliquefaciens* RSUCC0282 could inhibit the growth of *Staphylococcus aureus* ATCC25923. Furthermore, the cocultivation and HPLC analyses showed that the extracts prepared from 3 pairs of culture between *Staphylococcus* sp. RSUCC0020, *Micrococcus luteus* RSUCC0053, *Staphylococcus* sp. RSUCC0087, and *Staphylococcus pasteuri* RSUCC0090 could inhibit the growth of *Staphylococcus aureus* ATCC25923 and produced distinct chemical profiles from their single culture condition. Our study led to the isolation and identification of several promising bacterial strains for production of secondary metabolites that might be useful in biomedical applications.

## 1. Introduction

There are several problems around the world including suspension of petroleum energy source, unresolved pollution, climate change, emerging diseases, untreated diseases, and drug resistance pathogens. We direct our expectation toward the natural resources, which might possess unexplored natural compounds, to combat the mentioned problems [1–4]. Considering the natural compounds, the diverse chemical structures of secondary metabolites produced by microorganisms were proposed to be the valuable metabolites that act against the harmful biochemicals or proteins. Among the microbes, the easily evolving bacteria especially possess the diverse ability to produce the diverse secondary metabolites through their biosynthesis pathways. Genome-wide studies revealed that the bacteria species could synthesize groups of secondary metabolites including nonribosomal peptides (NRP), polyketides (PK), terpene (TP), and hybrid PK/NRP [5–7]. The bacteria use secondary metabolite as biological weapon against the other species in the same niche; protect themselves from physical environment, communication substances, and symbiosis stimulant, or even are hormones or pheromones to other organisms. Accordingly, novel secondary metabolites identified from the unexplored bacterial strains might be used as beneficial compounds for both industry and medical applications [8, 9].

In order to isolate a new bacterial strain from natural specimen of interest, we need appropriate protocols for isolation, identification, and maintenance of the bacterial strains from the natural resources. These include the use of appropriate isolation media depending on the niche of the bacteria target to promote and accelerate the growth of the bacterial strain but not from the standard media. Afterward, standard PCR is used for amplifying 16S ribosomal RNA gene fragment from bacterial isolated genomic DNA. Subsequently, the PCR product is then sequenced and compared with the 16S ribosomal RNA gene of the closely related bacterial strains using phylogenetic analysis [10]. By using degenerate primer pairs designed for the specific amplification of each class of the genes related to the biosynthesis of secondary metabolites, the number of gene fragments reflects the potential of the bacteria to synthesize the secondary metabolites for their own benefits [11]. Consequently, the new identified species with high number of genes that might be involved in the biosynthesis of secondary metabolites exhibit more chance to produce unidentified secondary metabolites with novel functions or bioactivities. Not only the degenerate PCR amplification but also the genome sequence data mining became the new era for the discovery of new genes that might be involved in the biosynthesis of new secondary metabolite from bacterial species. There are enormous numbers of genes identified from the genome sequence data up to date; however, only few of them were characterized for their biosynthetic functions [12–14]. The new identified species with high secondary metabolite production potential genes might not produce any bioactive compound in the standard laboratory condition, that is, the normal situation found in several studies. This is because the genes that might be involved in secondary metabolism are not expressed in the standard culture condition. Therefore, culture condition optimization or genetic engineering processes are needed for activation of the biosynthesis pathway [15]. A study in 2017 showed that a simple cocultivation technique between new identified bacteria with a fungus could stimulate the production of surfactant from the unproduced single culture condition. The explanation behind the situation would be the physiological response of the bacteria to the competitor in the same niche. The cocultivation technique should be also considered for the analysis of bioactivity of the newly identified bacterial strains [16].

There are only 15% of bacterial strains in the world that were isolated, identified, and used in biotechnological laboratory. In particular, Thailand, which is located in an appropriate residential zone for bacterial strains, might be rich in unexplored natural bacterial resources such as the sea, soil, and forest. Accordingly, unidentified secondary metabolites from the bacterial species should be explored as well [17]. Accordingly, in this work, we aim to identify new bacterial strains with high secondary metabolite production potential from our natural resources including seawater, soil, forest wood, and herbs. Furthermore, we also aim to identify their ability to produce compounds that might be useful in medical application using both single standard culture condition and cocultivation culture condition. We expect that the new identified species with high secondary metabolite production potential would be a promising bacterial strain that could produce biomedical valuable secondary metabolite.

## 2. Materials and Methods

### 2.1. Isolation and Maintenance of Bacterial Strains

The natural specimens consisting of 100 mg of Bangkhuntien's mangrove forest soil, Nam Nao National Park's soil, and Yaowarat Chinese market herbs were mixed with sterile distilled water to final volume of 10 mL. For Rayong's seawater, 10 mL was collected and diluted 1,000 times with NSS before use. After homogenization, 100 *µ*L of the aqueous solutions was plated on agar plate of CDA, MEA, MRS, NA, PCA, PDA, SDA, and TSA (Difco, USA). We incubated the plate 3 days and then transferred single colony into new NA agar plate. During doing bacteria culture, bacterial Gram of all strains was identified by standard Gram staining. In this study, we used 4 importance human pathogens as standards for antibacterial assay, namely, *Bacillus cereus* ATCC14579, *Escherichia coli* ATCC25922, *Pseudomonas aeruginosa* ATCC27853, and *Staphylococcus aureus* ATCC25923 (ATCC, USA). In order to maintain all the isolated and standard bacterial strains, we kept their 3-day NB cultures with 25% glycerol and restocked them every 6 months at −80*°*C. All bacteria strains' genomic DNA was prepared using Presto™ Mini gDNA Bacteria Kit following the manufacturer protocol (Geneaid, Taiwan).

### 2.2. Phylogenetic Analysis of Amplified 16S rRNA Gene Fragments

We used 16S27F (5′-AGAGTTTGATCCTG GCTCAG-3′) and 16S1492R (5′-GGTTACCTTGTTACGACTT-3′) as primers for amplification of 16S rRNA genes from all isolated bacterial genomic DNA [18]. A PCR reaction consists of 10 ng genomic DNA, 1X PCR buffer, 2.5 mM MgCl_2_, 1 mM dNTP, and 1 unit of DNA polymerase (Biotechrabbit, Germany). We used the initial denaturation at 94°C for 2 min, followed by 35 rounds of 94°C for 30 sec, 54°C for 30 sec, and 72°C for 2 min before final extension at 72°C for 5 min as thermal cycler condition. The amplified DNA fragments were then purified using GenepHlow Gel/PCR Kit (Geneaid, Taiwan), and subsequently their DNA sequence was determined via sequencing service of SolGent company (SolGent, Korea). The DNA sequence data were initially compared with NCBI database using BLAST. DNA of closely related species was then retrieved from the database; we used ClustalX for multiple sequence alignment and then reconstructed phylogenetic trees by programs in PHYLIP package. The unrooted phylogenetic trees were constructed using neighbor-joining method under Kimura 2-parameter model. Newick's standard files were visualized by FigTree v1.3.1. Bootstrap values obtained from 1,000 replicate pseudosamples were overlaid onto the visualized phylogenetic trees in percentages of the consensus.

### 2.3. PCR Amplification of Secondary Metabolism Gene Fragments

Diversity of NRPS, PKS, and TPS genes among isolated bacterial genomes was inspected using previously designed degenerate primer pairs. MTF2 (5′-GCNGGYGGYGCNTAYGTNCC-3′) and MTR (5′-CCNCGDATYTTNACYTG-3′) (annealing temperature at 45°C) were used for amplification of A domain of NRPS gene [6]. KS-F (5′-CGCTCCATGGAYCCSCARCA-3′) and KS-R (5′-GTCCCGGTGCCRTGSSHYTCSA-3′) (annealing temperature at 50°C) were used for amplification of KS domain of PKS gene [7]. TerpCyc/fw/ (5′-ACTGGTAYGTBTGGGTBTTCT-3′) and TerpCyc/rv/ (5′-SRCVGTGTKCTCGAACTCSTG-3′) (annealing temperature at 46°C) were used for amplification of cyclase TPS gene [5]. Each reaction containing genomic 10 ng of DNA, 1X PCR buffer, 2.5 mM MgCl_2_, 1 mM dNTP, and 1 unit DNA of polymerase (Biotechrabbit, Germany) was amplified using thermal cycler under the following conditions: initial denaturation at 94°C for 2 min; 35 rounds of 94°C for 30 sec; annealing temperature according to the pair of primers for 30 sec; 72°C for 2 min, before final extension at 72°C for 5 min.

### 2.4. Crude Extract Preparation from Single Cultivation and Cocultivation

A single colony of each bacterial strain was used for inoculum preparation. A 5 mL NB overnight culture was then diluted with NSS until the turbidity reached McFarland's standard No. 0.5 (approximately 1.5 × 10^8^ cells/mL). One mL of the inoculum was then added to 100 mL of freshly prepared NB media and further incubated at 37°C for 7 days. For cocultivation, 2 of the inoculums were added to the same 100 mL NB media and further incubated at the same condition as the single cultivation. After that, the cultured media were then collected by centrifugation and extracted by equal volume of ethyl acetate in separation funnel, and the ethyl acetate fraction was then collected. Subsequently, we used rotary evaporator to evaporate the collected fractions into the crude extract. The crude extract of each bacterial strain was prepared into triplicates.

### 2.5. Disc Diffusion Assay

*Bacillus cereus* ATCC14579, *Escherichia coli* ATCC25922, *Pseudomonas aeruginosa* ATCC27853, and *Staphylococcus aureus* ATCC25923 (ATCC, USA) were used as pathogenic representatives for antibacterial screening in this work. The NSS inoculum of each standard bacterium with approximately 1.5 × 10^8^ cells/mL was swabbed on NA. Five mg of each prepared crude extract was impregnated into sterile 6 mm diameter filter paper discs (Whatman, UK) before being placed onto the swabbed NB. The discs containing ampicillin (10 *μ*g/disc), ceftriaxone (30 *μ*g/disc), chloramphenicol (30 *μ*g/disc), and rifampicin (5 *μ*g/disc) (Oxoid, UK) were used as positive control for *Escherichia coli*, *Pseudomonas aeruginosa*, *Bacillus cereus*, and *Staphylococcus aureus*, respectively. After incubation for overnight, the antibacterial activity of each crude extract was determined by measuring the IZD in mm.

### 2.6. HPLC Analysis

In this work, we used UltiMate 3000 UHPLC systems (Thermo Scientific, USA) equipped with VertiSep reverse phase column C18 (Vertichrom, Thailand) to analyze the chemical profile of all 100 *μ*g/mL in acetonitrile crude extracts. The initial observed wavelengths of the photodiode array were set at 210 and 254 nm. We used water (A) and acetonitrile (B) as mobile phase together with the following machine conditions to separate the chemical constituents of the crude extracts: 0–20 minutes, 5% B; 20–30 minutes, from 5 to 50% B; 30–50 minutes, 50% B; 50–60 minutes, 50–80% B; 60–80 minutes, 80% B; 80–90 minutes, from 80 to 100% B; 90–120 minutes, 100% B; 120–140 minutes, from 100 to 5% B. The column was equilibrated for 140–155 minutes with 5% B before the next run.

## 3. Results

### 3.1. Diversity of Bacterial Strains Isolated from Thailand's Natural Resources

**Figure 1:**
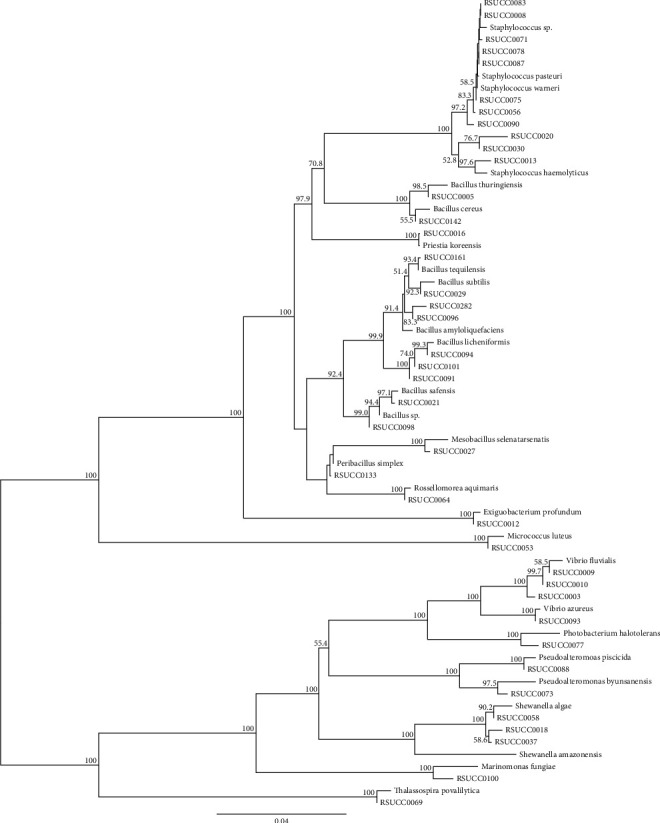
Phylogenetic tree constructed from 16S rRNA gene fragments of 40 isolated bacterial strains together with known close evolutionary relationship bacterial species.

We could isolate 40 morphological distinct bacterial strains from Thailand's 4 natural sources: 16 strains from Bangkhuntien's mangrove forest soil, 1 strain from Nam Nao National Park's soil, 20 strains from Rayong's seawater, and 3 strains from Yaowarat Chinese market herbs. After we sent and obtained the data of approximately 1,400 bp of the 40 strains' amplified 16S rRNA gene fragments, we could assign the putative species of all the strains using general basic local alignment search tool (BLAST) to search and prepare phylogenetic analysis. BLAST analysis showed that their 16S rRNA sequences had over 98% similarity with the sequence of known species stored in National Center for Biotechnology Information (NCBI) database. The phylogenetic analysis with high confidential value constructed from 1,000 pseudosamples also supported the correct identification of the species (Figure 1). There are 8 strains that were identified as putative pathogenic bacteria: *Bacillus cereus* RSUCC0142, *Bacillus subtilis* RSUCC0029, *Staphylococcus haemolyticus* RSUCC0013, *Staphylococcus pasteuri* RSUCC0056, *Staphylococcus pasteuri* RSUCC0090, *Vibrio fluvialis* RSUCC0003, *Vibrio fluvialis* RSUCC0009, and *Vibrio fluvialis* RSUCC0010. The highest diverse genus isolated from the 4 resources in our study is *Bacillus*, which accounted for 37.5% of total isolated strains. We could isolate more Gram-positive bacteria (28 strains) than Gram-negative bacteria (12 strains) (Table 1).

### 3.2. Diversity of NRPS, PKS, and TPS Genes in Isolated Bacteria

Degenerate PCR amplification by NRPS primers from DNA of 40 isolated bacteria yielded the highest number of PCR products among the 3 classes of secondary metabolism genes. Furthermore, the amplification of NRPS A domain showed that there are 31 of all bacteria strains containing NRPS genes within their genomes. For KS domain of PKS genes, PCR amplification gave positive results from 22 bacteria strains' genomic DNA. Among the 22 bacterial strains, 11 were the bacteria in genus *Bacillus*. Surprisingly, we could not amplify KS domain from 10 strains of genus *Staphylococcus*. We could amplify TPS from 29 isolated bacterial strains' genomic DNA, which included both Gram-positive and Gram-negative bacterial strains. Interestingly, we could not amplify TPS from genomic DNA of *Pseudoalteromonas byunsanensis* RSUCC0073, *Pseudoalteromonas piscicida* RSUCC0088, *Vibrio azureus* RSUCC0093, and *Marinomonas fungiae* RSUCC0100, which are Gram-negative bacteria. The strain that possesses the highest number of amplified secondary metabolism gene fragments in this study is *Bacillus licheniformis* RSUCC0101. We could amplify 26 PCR products from its genomic DNA using 3 primer pairs consisting of 6 NRPS A domains, 9 PKS KS domains, and 11 TPS cyclases (Table 2) (Supplementary Materials 1–3).

### 3.3. Bioactivity and Metabolite Profile of the Extracts Prepared from Single Culture and Coculture Media

**Figure 2:**
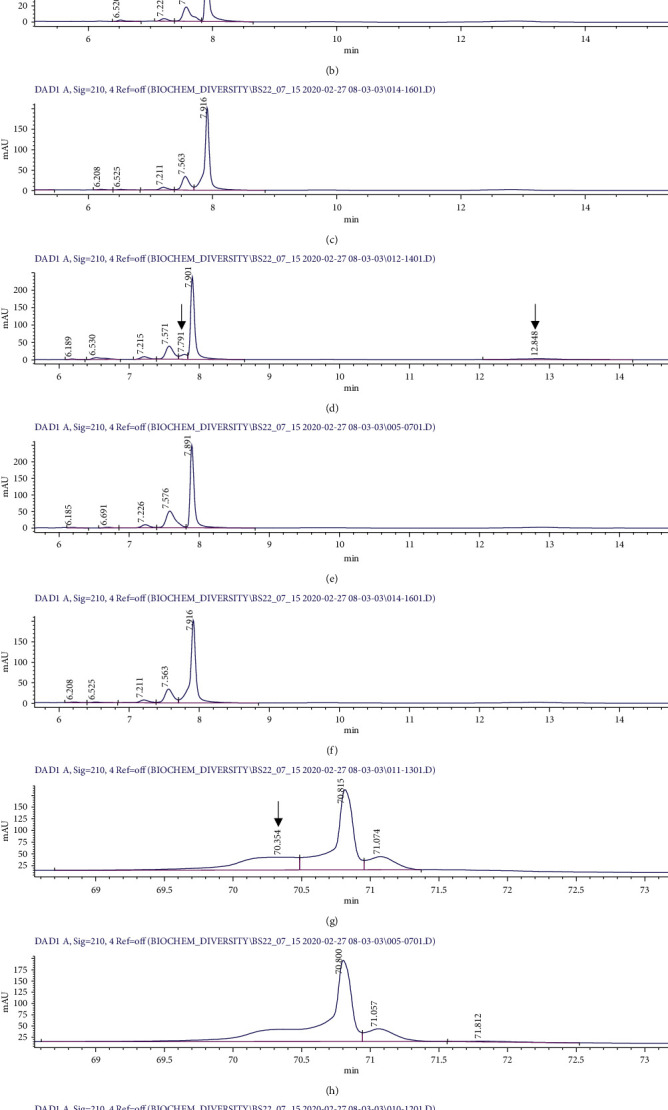
HPLC analyses of the bioactive coculture extracts together with their corresponding single culture extracts; *A* = 20-53, *B* = 20, *C* = 53, *D* = 53-90, *E* = 53, *F* = 90, *G* = 87-90, *H* = 87, *I* = 90; extra metabolite signals detected by the detector are indicated by arrows.

Crude extracts prepared from media of single culture of *Bacillus safensis* RSUCC0021 and *Bacillus amyloliquefaciens* RSUCC0282 could inhibit the growth of *Staphylococcus aureus* ATCC25923 with IZD values of 8.0 ± 1.30 and 19.0 ± 1.50 mm, respectively (Supplementary Material 4). However, the extracts from the two bacterial strains could not inhibit the growth of *Bacillus cereus* ATCC14579, *Escherichia coli* ATCC25922, and *Pseudomonas aeruginosa* ATCC27853. Surprisingly, the extracts prepared from coculture experiments between *Bacillus safensis* RSUCC0021 or *Bacillus amyloliquefaciens* RSUCC0282 and other isolated bacterial strains could not inhibit any of 4 standard pathogenic bacteria. Furthermore, the 3 extracts prepared from 3 pairs of coculture between *Staphylococcus* sp. RSUCC0020 and *Micrococcus luteus* RSUCC0053 (20-53), *Micrococcus luteus* RSUCC0053 and *Staphylococcus pasteuri* RSUCC0090 (53-90), and *Staphylococcus* sp. RSUCC0087 and *Staphylococcus pasteuri* RSUCC0090 (87-90) could inhibit the growth of *Staphylococcus aureus* ATCC25923 with IZD values of 9.3 ± 0.57, 7.7 ± 0.57, and 9.7 ± 0.57 mm, respectively (Table 3) (Supplementary Material 5). Average IZD value of rifampicin against *Staphylococcus aureus* ATCC25923 of the whole experiments is 29.4 ± 1.17 mm. We observed the change of bioactivity; however, only small change of metabolite profiles was observed between the single culture and coculture extracts. These included very low extra metabolite peaks present in coculture extracts of 20-53, 53-90, and 87-90 but absent in their single culture extracts at retention time of 6.7, 7.8, and 70.3 minutes, respectively (Figure 2).

## 4. Discussion

Bacteria are the largest diverse microorganisms found in the world. They have a major role as the digester in the ecosystem. They transform macromolecules into small metabolites that can be absorbed easier by other organisms in the same niche. In order to maintain their relationship with the environment, bacteria might produce secondary metabolites as antibiotics, protectants, stimulants, hormones, and pheromones [19]. Because of the unexplored bacterial strains and their secondary metabolite diversity, the undiscovered bacteria in Thailand's natural resources might be a promising source of new bioactive secondary metabolites [17]. Consequently, we initiated our project to explore, isolate, and identify high potential secondary metabolite producing bacterial strains from Bangkhuntien's mangrove forest soil, Nam Nao National Park's soil, Rayong's seawater, and Yaowarat Chinese market herbs. We successfully isolated and identified 40 bacterial strains from the mentioned Thailand resources. Using general BLAST search and phylogenetic analysis, we assigned the genus and species names to all of the strains (Supplementary Material 6). We could identify more Gram-positive (28 strains) than Gram-negative (12 strains) bacteria from the natural resources similar to the study in 2014. The ratios of bacteria diversity of the current study were 70% and 30%, but 61% and 39% in the previous study, for Gram-positive and Gram-negative bacteria, respectively [20]. The Gram-positive bacteria usually play the major role in carbon, nitrogen, and phosphorus cycle. Accordingly, this might be the reason why we could isolate diverse Gram-positive bacterial strains from the sea, soil, and tree resources [21].

We could amplify A domain of NRPS from 22 bacterial strains and KS domain of PKS gene from 31 bacterial strains, respectively. Interestingly, half of them that give positive PCR amplification results belong to *Bacillus*. It is well known that *Bacillus* genus is defined as high potential NRP, PK, and hybrid PK/NRP producer in previous genome mining [22]. Especially for our strain, *Bacillus licheniformis* RSUCC0101, its genome should contain very high secondary metabolism gene paralogs for production of NRP (6 A domains), PK (9 KS domains), and TP (11 cyclases) revealed by our degenerate PCR amplifications. The result showed the potential of the strain to be a high potent secondary metabolite producer similar to previous bioinformatics genome mining study [23]. However, the amplification of KS domain from *Staphylococcus* species yielded negative PCR products except *Staphylococcus* sp. RSUCC0008. We hypothesized that the positive gene fragment amplification from *Staphylococcus* sp. RSUCC0008 could be a result of horizontal gene transfer from the other bacterial species within the same niche [24]. This hypothesis should be proven by further full-length PKS gene amplification, gene sequencing, genome sequencing, and phylogenetic analysis. For TPS gene amplification, we expected higher TPS gene diversity in Gram-negative than Gram-positive bacteria, which was discussed previously in 2015 [25]. However, our results showed no significant difference in TPS gene diversity between Gram-positive and Gram-negative bacteria. Furthermore, we could not amplify TPS gene fragments from *Pseudoalteromonas byunsanensis* RSUCC0073, *Pseudoalteromonas piscicida* RSUCC0088, *Vibrio azureus* RSUCC0093, and *Marinomonas fungiae* RSUCC0100.

*Bacillus safensis* was first isolated from Mars Odyssey Orbiter spacecraft. It was reported to synthesize several carotene terpenoid substances for protecting its cell from UV irradiation [26]. In our work, the extract prepared from single culture of *Bacillus safensis* RSUCC0021 could inhibit the growth of *Staphylococcus aureus* ATCC25923 with IZD value of 8.0 ± 1.30 mm. This bioactivity should belong to NRP, PK, or hybrid PK/NRP rather than TP. Accordingly, we amplified 2 A domains of NRPS gene from the strains, which might be involved in the biosynthesis of the antibacterial compounds identified in 2014 [27]. The extract prepared from *Bacillus amyloliquefaciens* RSUCC0282 could also inhibit the growth of *Staphylococcus aureus* ATCC25923 with IZD value of 19.0 ± 1.50 mm. However, there is no previous report showing that *Bacillus amyloliquefaciens* could produce secondary metabolite compound against the growth of *Staphylococcus aureus*. *Bacillus amyloliquefaciens* was usually used as a biocontrol agent that can stimulate plant immune system against pathogenic fungi *Ralstonia solanacearum*, *Rhizoctonia solani*, and *Alternaria tenuissima*. Moreover, *Bacillus amyloliquefaciens* could produce barnase and NRP plantazolicin that specifically inhibit the growth of *Bacillus anthracis* [28]. The antibacterial activity of the extracts prepared in our study against *Staphylococcus aureus* would need further investigation for chemical structure elucidation of the bioactive compounds. Surprisingly, we could not observe bioactivity of any extracts prepared from coculture of *Bacillus safensis* RSUCC0021 and *Bacillus amyloliquefaciens* RSUCC0282 against *Staphylococcus aureus* ATCC25923. It is possible that the growth of *Bacillus safensis* RSUCC0021 and *Bacillus amyloliquefaciens* RSUCC0282 was inhibited and/or obtained inappropriate signal from the pairing strains under coculture condition. Consequently, there were no productions of the antibacterial agents from them via growth inhibition and/or stimulation.

From all 780 extracts prepared from coculture between the 40 isolated strains in our study, only 3 extracts exhibited antibacterial activity against *Staphylococcus aureus* ATCC25923. These are the extracts prepared from 20-53, 53-90, and 87-90. We tried to observe the differences of secondary metabolite profile between the 2 coculture extracts compared with their single culture extracts using HPLC analyses; however, only 3 small peaks referring to 3 extra metabolites were identified. Furthermore, there was no correlation between the 3 extra metabolites that were observed in the HPLC profile from the coculture of *Staphylococcus* sp. RSUCC0020, *Micrococcus luteus* RSUCC0053, *Staphylococcus* sp. RSUCC0087, and *Staphylococcus pasteuri* RSUCC0090. According to the strain evolutionary tree, we hypothesized that the production of the 3 extra metabolites might be a result of food source competition. They produced antibacterial compound that can inhibit the growth of *Staphylococcus aureus*, in which they have closely evolutionary relationship with. The production of bioactive compound for food source competition in coculture condition might be similar to the previous report in 2017 [16]. The 3 extra metabolites should be further isolated and purified and undergo chemical structure elucidation.

## 5. Conclusion

We conducted our work with the aim of isolating and identifying high secondary metabolite production potential bacterial strains from Thailand's natural resources. We used degenerate PCR genome mining together with antibacterial bioactivity analysis of the extracts prepared from their single culture and coculture condition for selection of the high potent strains. According to the PCR amplification, we found that the highest potent strain is *Bacillus licheniformis* RSUCC0101 whose genome has at least 6 A domains of NRPS, 9 KS domains of PKS, and 11 cyclases of TPS. The single culture extracts prepared from *Bacillus safensis* RSUCC0021 and *Bacillus amyloliquefaciens* RSUCC0282 could inhibit the growth of *Staphylococcus aureus* ATCC25923. Consequently, they were identified as high potential strains for production of antibacterial agents that need further investigation of the secondary metabolites in their chemical profiles. The coculture experiments results showed that *Staphylococcus* sp. RSUCC0020, *Micrococcus luteus* RSUCC0053, *Staphylococcus* sp. RSUCC0087, and *Staphylococcus pasteuri* RSUCC0090 could differently produce extra 3 secondary metabolites from their single culture extracts. The 3 metabolites could inhibit the growth of *Staphylococcus aureus* ATCC25923, so further chemical investigation and spectroscopic analyses of their chemical structures in future work are needed.

## Figures and Tables

**Table 1:** Forty bacterial strains isolated and identified from Thailand's natural resources in the current study.

Bacteria genus and species	RSUCC	Gram	Source	16S rRNA GenBank accession number
*Vibrio fluvialis*	0003	−	Rayong's seawater	OK056286
*Bacillus thuringiensis*	0005	+	Rayong's seawater	OK056287
*Staphylococcus* sp.	0008	+	Rayong's seawater	OK056288
*Vibrio fluvialis*	0009	−	Rayong's seawater	OK056289
*Vibrio fluvialis*	0010	−	Rayong's seawater	OK056290
*Exiguobacterium profundum*	0012	+	Rayong's seawater	OK056291
*Staphylococcus haemolyticus*	0013	+	Rayong's seawater	OK056292
*Priestia koreensis*	0016	+	Rayong's seawater	OK056293
*Shewanella haliotis*	0018	−	Rayong's seawater	OK056294
*Staphylococcus* sp.	0020	+	Rayong's seawater	OK056295
*Bacillus safensis*	0021	+	Rayong's seawater	OK056296
*Mesobacillus selenatarsenatis*	0027	+	Rayong's seawater	OK056297
*Bacillus subtilis*	0029	+	Rayong's seawater	OK056298
*Staphylococcus* sp.	0030	+	Rayong's seawater	OK056299
*Shewanella algae*	0037	−	Rayong's seawater	OK056300
*Micrococcus luteus*	0053	+	Rayong's seawater	OK056301
*Staphylococcus pasteuri*	0056	+	Rayong's seawater	OK056302
*Shewanella amazonensis*	0058	−	Rayong's seawater	OK056303
*Bacillus aquimaris*	0064	+	Rayong's seawater	OK056304
*Thalassospira povalilytica*	0069	−	Rayong's seawater	OK056305
*Staphylococcus* sp.	0071	+	Bangkhuntien's soil	OK056306
*Pseudoalteromonas byunsanensis*	0073	−	Bangkhuntien's soil	OK056307
*Staphylococcus warneri*	0075	+	Bangkhuntien's soil	OK056308
*Photobacterium halotolerans*	0077	−	Bangkhuntien's soil	OK056309
*Staphylococcus* sp.	0078	+	Bangkhuntien's soil	OK056310
*Staphylococcus* sp.	0083	+	Bangkhuntien's soil	OK056311
*Staphylococcus* sp.	0087	+	Bangkhuntien's soil	OK056312
*Pseudoalteromonas piscicida*	0088	−	Bangkhuntien's soil	OK056313
*Staphylococcus pasteuri*	0090	+	Bangkhuntien's soil	OK056314
*Bacillus licheniformis*	0091	+	Bangkhuntien's soil	OK056315
*Vibrio azureus*	0093	−	Bangkhuntien's soil	OK056316
*Bacillus licheniformis*	0094	+	Bangkhuntien's soil	OK056317
*Bacillus licheniformis*	0096	+	Bangkhuntien's soil	OK056318
*Bacillus* sp.	0098	+	Bangkhuntien's soil	OK056319
*Marinomonas fungiae*	0100	−	Bangkhuntien's soil	OK056320
*Bacillus licheniformis*	0101	+	Bangkhuntien's soil	OK056321
*Peribacillus simplex*	0133	+	Yaowarat's herbs	OK056322
*Bacillus cereus*	0142	+	Yaowarat's herbs	OK056323
*Bacillus tequilensis*	0161	+	Yaowarat's herbs	OK056324
*Bacillus amyloliquefaciens*	0282	+	Nam Nao Park's soil	OK056325

**Table 2:** Number of secondary metabolism gene fragments amplified from degenerate PCR of the 40 isolated bacterial strains overlaid with their cladogram.

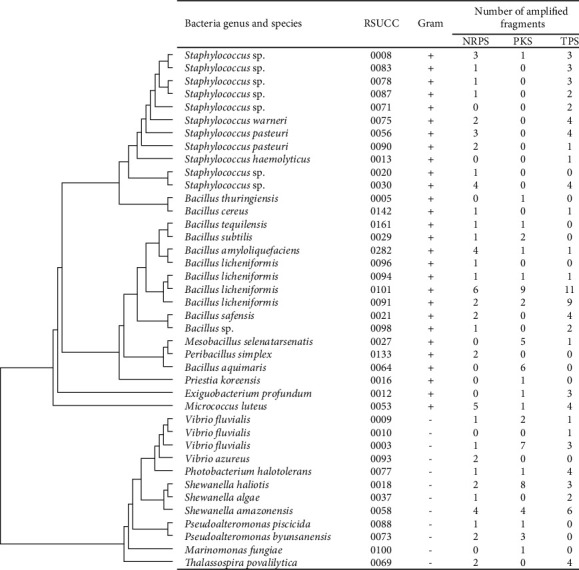

**Table 3:** IZD values of the crude extracts prepared from 6 bacterial strains that could inhibit the growth of *Staphylococcus aureus* ATCC25923 in single culture or coculture experiments.

Bacterial strains and IZD values	*Staphylococcus* sp. RSUCC0020	*Bacillus safensis* RSUCC0021	*Micrococcus luteus* RSUCC0053	*Staphylococcus* sp. RSUCC0087	*Staphylococcus pasteuri* RSUCC0090	*Bacillus amyloliquefaciens* RSUCC0282
*Staphylococcus* sp. RSUCC0020	ND					
*Bacillus safensis* RSUCC0021	ND	8.0 ± 1.30				
*Micrococcus luteus* RSUCC0053	9.3 ± 0.57	ND	ND			
*Staphylococcus* sp. RSUCC0087	ND	ND	ND	ND		
*Staphylococcus pasteuri* RSUCC0090	ND	ND	7.7 ± 0.57	9.7 ± 0.57	ND	
*Bacillus amyloliquefaciens* RSUCC0282	ND	ND	ND	ND	ND	19.0 ± 1.50

ND: not determined or regarded as zero. Average IZD of rifampicin (positive control) is 29.4 ± 1.17.

## Data Availability

The datasets used and/or analyzed during the current study are available from the corresponding author on reasonable request.

## Abbreviations

A domain: Adenylation domain
ATCC: American type culture collection
BLAST: Basic local alignment search tool
CDA: Czapek Dox agar
DMSO: Dimethyl sulfoxide
HPLC: High performance liquid chromatography
IZD: Inhibition zone diameter
KS domain: Ketosynthase domain
MEA: Malt extract agar
MRS: De Man, Rogosa, and Sharpe agar
NA: Nutrient agar
NB: Nutrient broth
NRP: Nonribosomal peptide
NRPS: Nonribosomal peptide synthetase
NSS: Normal saline solution
PCA: Plate count agar
PCR: Polymerase chain reaction
PK: Polyketide
PKS: Polyketide synthase
RSUCC: Rangsit University culture collection
SDA: Sabouraud dextrose agar
TP: Terpene
TPS: Terpene synthase
TSA: Trypticase soy agar.

## Supplementary Materials

Supplementary Materials Supplementary Material 1: gel figures of NRPS PCR fragments. Supplementary Material 2: gel figures of PKS PCR fragments. Supplementary Material 3: gel figures of TPS PCR fragments. Supplementary Material 4: positive disc diffusion assay figures of extracts prepared from single cultures. Supplementary Material 5: positive disc diffusion assay figures of extracts prepared from cocultures. Supplementary Material 6: the list of accession numbers of all 16S rRNA sequences used for reconstructing phylogenetic tree in our work.

## References

[1] Chen M., Xu P., Zeng G., Yang C., Huang D., Zhang J. (2015). Bioremediation of soils contaminated with polycyclic aromatic hydrocarbons, petroleum, pesticides, chlorophenols and heavy metals by composting: applications, microbes and future research needs. *Biotechnology Advances*.

[2] Enserink M. (2004). A global fire brigade responds to disease outbreaks. *Science*.

[3] Iwaro J., Mwasha A. (2010). A review of building energy regulation and policy for energy conservation in developing countries. *Energy Policy*.

[4] Pogue J. M., Kaye K. S., Cohen D. A., Marchaim D. (2015). Appropriate antimicrobial therapy in the era of multidrug-resistant human pathogens. *Clinical Microbiology and Infections*.

[5] Kim J. (2011). Natural product biosynthesis in uncultured bacteria, Student Theses and Dissertations. http://hdl.handle.net/10209/392.

[6] Neilan B. A., Dittmann E., Rouhiainen L. (1999). Nonribosomal peptide synthesis and toxigenicity of cyanobacteria. *Journal of Bacteriology*.

[7] Song J. W., Dong X. Y., Jiao B. H., Wang L. H. (2013). Directly accessing the diversity of bacterial type I polyketide synthase gene in Chinese soil and seawater. *African Journal of Microbiology Research*.

[8] Brunati M., Rojas J. L., Sponga F. (2009). Diversity and pharmaceutical screening of fungi from benthic mats of Antarctic lakes. *Marine Genomics*.

[9] Mousa W. K., Raizada M. N. (2013). The diversity of anti-microbial secondary metabolites produced by fungal endophytes: an interdisciplinary perspective. *Frontiers in Microbiology*.

[10] Phumudzo T., Ronald N., Khayalethu N., Fhatuwani M. (2013). Bacterial species identification getting easier. *African Journal of Biotechnology*.

[11] Nicholson T. P., Rudd B. A. M., Dawson M., Lazarus C. M., Simpson T. J., Cox R. J. (2001). Design and utility of oligonucleotide gene probes for fungal polyketide synthases. *Chemistry & Biology*.

[12] Donadio S., Monciardini P., Sosio M. (2007). Polyketide synthases and nonribosomal peptide synthetases: the emerging view from bacterial genomics. *Natural Product Reports*.

[13] Omura S., Ikeda H., Ishikawa J. (2001). Genome sequence of an industrial microorganism *Streptomyces avermitilis*: deducing the ability of producing secondary metabolites. *Proceedings of the National Academy of Sciences*.

[14] Weber T., Blin K., Duddela S. (2015). antiSMASH 3.0-a comprehensive resource for the genome mining of biosynthetic gene clusters. *Nucleic Acids Research*.

[15] Bergmann S., Schumann J., Scherlach K., Lange C., Brakhage A. A., Hertweck C. (2007). Genomics-driven discovery of PKS-NRPS hybrid metabolites from *Aspergillus nidulans*. *Nature Chemical Biology*.

[16] Wakefield J., Hassan H. M., Jaspars M., Ebel R., Rateb M. E. (2017). Dual induction of new microbial secondary metabolites by fungal bacterial co-cultivation. *Frontiers in Microbiology*.

[17] Hawksworth D. L., Watling R., Frankland J. C., Ainsworth A. M., Issac S., Robinson C. H. (2002). Why study tropical fungi?. *Tropical Mycology*.

[18] Jeganathan P., Rajasekaran K. M., Asha Devi N. K., Karuppusamy S. (2013). Antimicrobial activity and characterization of marine bacteria. *Indian Journal of Pharmaceutical and Biological Research*.

[19] Demain A. L., Fang A. (2000). The natural functions of secondary metabolites. *Advances in Biochemical Engineering*.

[20] Al-Zereini W. A. (2014). Bioactive crude extracts from four bacterial isolates of marine sediments from Red Sea, gulf of Aqaba, Jordan. *Jordan Journal of Biological Sciences*.

[21] Yilmaz M., Soran H., Beyatli Y. (2006). Antimicrobial activities of some *Bacillus* spp. strains isolated from the soil. *Microbiological Research*.

[22] Zhao J. F., Zhang C., Lu J., Lu Z. X. (2016). Enhancement of fengycin production in *Bacillus amyloliquefaciens* by genome shuffling and relative gene expression analysis using RT-PCR. *Canadian Journal of Microbiology*.

[23] Harwood C. R., Mouillon J. M., Pohl S., Arnau J. (2018). Secondary metabolite production and the safety of industrially important members of the *Bacillus subtilis* group. *FEMS Microbiology Reviews*.

[24] Nivina A., Yuet K. P., Hsu J., Khosla C. (2019). Evolution and diversity of assembly-Line polyketide synthases. *Chemical Reviews*.

[25] Yamada Y., Kuzuyama T., Komatsu M. (2015). Terpene synthases are widely distributed in bacteria. *Proceedings of the National Academy of Sciences*.

[26] Khaneja R., Perez-Fons L., Fakhry S. (2010). Carotenoids found in *Bacillus*. *Journal of Applied Microbiology*.

[27] Domingos D. F., de Faria A. F., de Souza Galaverna R. (2015). Genomic and chemical insights into biosurfactant production by the mangrove-derived strain *Bacillus safensis* CCMA-560. *Applied Microbiology and Biotechnology*.

[28] Molohon K. J., Melby J. O., Lee J. (2011). Structure determination and interception of biosynthetic intermediates for the plantazolicin class of highly discriminating antibiotics. *ACS Chemical Biology*.

