# Supplementary material for: Secondary Metabolism Gene Diversity and Cocultivation toward Isolation and Identification of Potent Bioactive Compounds Producing Bacterial Strains from Thailand's Natural Resources
Source: Scientifica (Cairo). 2022 May 29;2022:2827831. doi: 10.1155/2022/2827831 (PMC9168185; doi:10.1155/2022/2827831)
Supplement: Supplementary Materials — Supplementary Material 1: gel figures of NRPS PCR fragments. Supplementary Material 2: gel figures of PKS PCR fragments. Supplementary Material 3: gel figures of TPS PCR fragments. Supplementary Material 4: positive disc diffusion assay figures of extracts prepared from single cultures. Supplementary Material 5: positive disc diffusion assay figures of extracts prepared from cocultures. Supplementary Material 6: the list of accession numbers of all 16S rRNA sequences used for reconstructing phylogenetic tree in our work. [file 2827831.f1.zip › 2827831.f1/Supplementary material 5.docx]

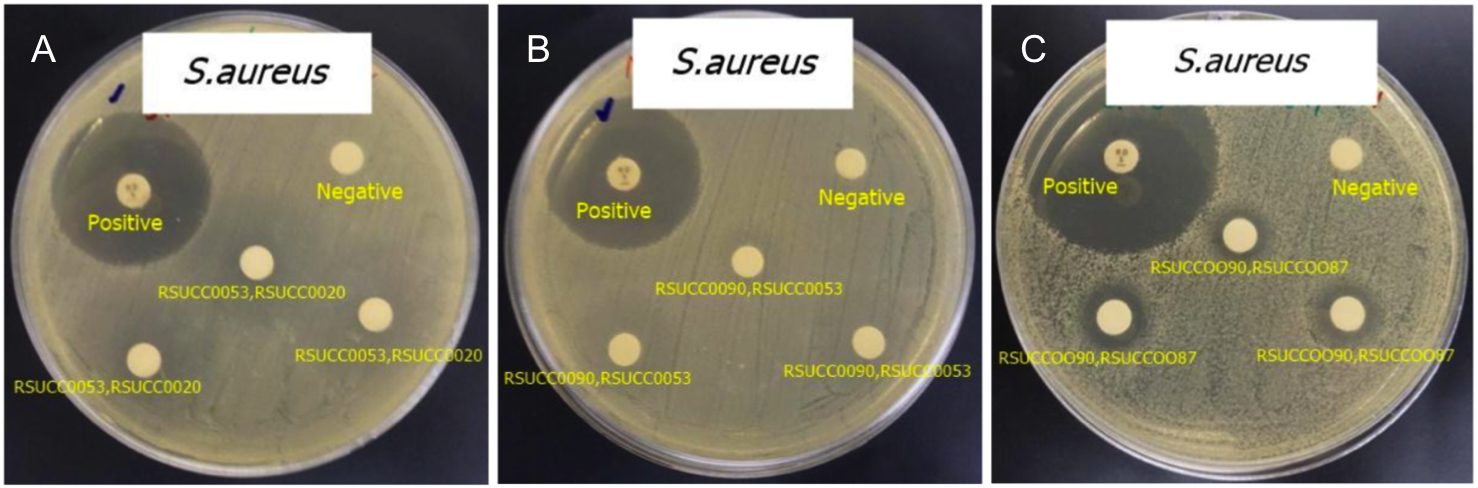


Supplementary material 5: Disc diffusion assay of extracts prepared from co-culture between *Staphylococcus* sp. RSUCC0020 and *Micrococcus luteus* RSUCC0053 (20-53) (A), *Micrococcus luteus* RSUCC0053 and *Staphylococcus pasteuri* RSUCC0090 (53-90) (B), and *Staphylococcus* sp. RSUCC0087 and *Staphylococcus pasteuri* RSUCC0090 (87-90) (C) against *Staphylococcus aureus* ATCC25923
