# Supplementary material for: Secondary Metabolism Gene Diversity and Cocultivation toward Isolation and Identification of Potent Bioactive Compounds Producing Bacterial Strains from Thailand's Natural Resources
Source: Scientifica (Cairo). 2022 May 29;2022:2827831. doi: 10.1155/2022/2827831 (PMC9168185; doi:10.1155/2022/2827831)
Supplement: Supplementary Materials — Supplementary Material 1: gel figures of NRPS PCR fragments. Supplementary Material 2: gel figures of PKS PCR fragments. Supplementary Material 3: gel figures of TPS PCR fragments. Supplementary Material 4: positive disc diffusion assay figures of extracts prepared from single cultures. Supplementary Material 5: positive disc diffusion assay figures of extracts prepared from cocultures. Supplementary Material 6: the list of accession numbers of all 16S rRNA sequences used for reconstructing phylogenetic tree in our work. [file 2827831.f1.zip › 2827831.f1/Supplementary material 6.docx]

Supplementary material 6: Accession number of all 16S rRNA sequence used for reconstructing phylogenetic tree in our works

| Full bacteria genus, species, and strains | Name on tree | 16S rRNA genbank accession number |
| --- | --- | --- |
| *Bacillus amyloliquefaciens* HGGY-5 | *Bacillus amyloliquefaciens* | KR708866.1 |
| *Bacillus amyloliquefaciens* RSUCC0282 | RSUCC0282 | OK056325.1 |
| *Bacillus aquimaris* W-N-4-3-2 | *Rossellomorea aquimaris* | KU570365.1 |
| *Bacillus aquimaris* RSUCC0064 | RSUCC0064 | OK056304.1 |
| *Bacillus cereus* EPB2 1 | *Bacillus cereus* | JQ308547.1 |
| *Bacillus cereus* RSUCC0142 | RSUCC0142 | OK056323.1 |
| *Bacillus koreensis* B11 | *Priestia koreensis* | MN880411.1 |
| *Bacillus licheniformis* HT6 | *Bacillus licheniformis* | JN013191.1 |
| *Bacillus licheniformis* RSUCC0091 | RSUCC0091 | OK056315.1 |
| *Bacillus licheniformis* RSUCC0094 | RSUCC0094 | OK056317.1 |
| *Bacillus licheniformis* RSUCC0096 | RSUCC0096 | OK056318.1 |
| *Bacillus licheniformis* RSUCC0101 | RSUCC0101 | OK056321.1 |
| *Bacillus safensis* D38 | *Bacillus safensis* | KU922207.1 |
| *Bacillus safensis* RSUCC0021 | RSUCC0021 | OK056296.1 |
| *Bacillus selenatarsenatis* SF-1 | *Mesobacillus selenatarsenatis* | NR_041465.1 |
| *Bacillus simplex* R11 | *Peribacillus simplex* | KJ534532.1 |
| *Bacillus* sp. CS14 | *Bacillus* sp. | MT584789.1 |
| *Bacillus* sp*.* RSUCC0098 | RSUCC0098 | OK056319.1 |
| *Bacillus subtilis* Bs08 | *Bacillus subtilis* | KP055032.1 |
| *Bacillus subtilis* RSUCC0029 | RSUCC0029 | OK056298.1 |
| *Bacillus tequilensis* 7PJ-7 | *Bacillus tequilensis* | KR708845.1 |
| *Bacillus tequilensis* RSUCC0161 | RSUCC0161 | OK056324.1 |
| *Bacillus thuringiensis* | *Bacillus thuringiensis* | LC146717.1 |
| *Bacillus thuringiensis* RSUCC0005 | RSUCC0005 | OK056287.1 |
| *Exiguobacterium profundum* HNS008 | *Exiguobacterium profundum* | JN128242.1 |
| *Exiguobacterium profundum* RSUCC0012 | RSUCC0012 | OK056291.1 |
| *Marinomonas fungiae* AN44 | *Marinomonas fungiae* | NR_133775.1 |
| *Marinomonas fungiae* RSUCC0100 | RSUCC0100 | OK056320.1 |
| *Mesobacillus selenatarsenatis* RSUCC0027 | RSUCC0027 | OK056297.1 |
| *Micrococcus luteus* T2 | *Micrococcus luteus* | KY317958.1 |
| *Micrococcus luteus* RSUCC0053 | RSUCC0053 | OK056301.1 |
| *Peribacillus simplex* RSUCC0133 | RSUCC0133 | OK056322.1 |
| *Photobacterium halotolerans* BDH29 | *Photobacterium halotolerans* | KF933623.1 |
| *Photobacterium halotolerans* RSUCC0077 | RSUCC0077 | OK056309.1 |
| *Priestia koreensis* RSUCC0016 | RSUCC0016 | OK056293.1 |
| *Pseudoalteromonas byunsanensis* FR1199 | *Pseudoalteromonas byunsanensis* | DQ011289.2 |
| *Pseudoalteromonas byunsanensis* RSUCC0073 | RSUCC0073 | OK056307.1 |
| *Pseudoalteromonas piscicida* MCCB 201 | *Pseudoalteromonas piscicida* | KF880965.1 |
| *Pseudoalteromonas piscicida* RSUCC0088 | RSUCC0088 | OK056313.1 |
| *Shewanella algae* RSUCC0037 | RSUCC0037 | OK056300.1 |
| *Shewanella amazonensis* SB2B | *Shewanella amazonensis* | NR_074842.1 |
| *Shewanella amazonensis* RSUCC0058 | RSUCC0058 | OK056303.1 |
| *Shewanella haliotis* D4 | *Shewanella algae* | KF500918.1 |
| *Shewanella haliotis* RSUCC0018 | RSUCC0018 | OK056294.1 |
| *Staphylococcus haemolyticus* AU I3 | *Staphylococcus haemolyticus* | KY775491.1 |
| *Staphylococcus haemolyticus* RSUCC0013 | RSUCC0013 | OK056292.1 |
| *Staphylococcus pasteuri* 208 | *Staphylococcus pasteuri* | MH910312.1 |
| *Staphylococcus pasteuri* RSUCC0056 | RSUCC0056 | OK056302.1 |
| *Staphylococcus pasteuri* RSUCC0090 | RSUCC0090 | OK056314.1 |
| *Staphylococcus* sp. MT-Y-S2 | *Staphylococcus* sp. | KU671206.1 |
| *Staphylococcus* sp. RSUCC0008 | RSUCC0008 | OK056288.1 |
| *Staphylococcus* sp*.* RSUCC0020 | RSUCC0020 | OK056295.1 |
| *Staphylococcus* sp*.* RSUCC0030 | RSUCC0030 | OK056299.1 |
| *Staphylococcus* sp*.* RSUCC0071 | RSUCC0071 | OK056306.1 |
| *Staphylococcus* sp*.* RSUCC0078 | RSUCC0078 | OK056310.1 |
| *Staphylococcus* sp*.* RSUCC0083 | RSUCC0083 | OK056311.1 |
| *Staphylococcus* sp*.* RSUCC0087 | RSUCC0087 | OK056312.1 |
| *Staphylococcus warneri* DK131 | *Staphylococcus warneri* | MT642942.1 |
| *Staphylococcus warneri* RSUCC0075 | RSUCC0075 | OK056308.1 |
| *Thalassospira povalilytica* Zumi 95 | *Thalassospira povalilytica* | NR_125450.1 |
| *Thalassospira povalilytica* RSUCC0069 | RSUCC0069 | OK056305.1 |
| *Vibrio azureus* KNUSC1042 | *Vibrio azureus* | KP342198.1 |
| *Vibrio azureus* RSUCC0093 | RSUCC0093 | OK056316.1 |
| *Vibrio fluvialis* EP27-13-11 | *Vibrio fluvialis* | KX002034.1 |
| *Vibrio fluvialis* RSUCC0003 | RSUCC0003 | OK056286.1 |
| *Vibrio fluvialis* RSUCC0009 | RSUCC0009 | OK056289.1 |
| *Vibrio fluvialis* RSUCC0010 | RSUCC0010 | OK056290.1 |
